# Surveys of knowledge and awareness of antibiotic use and antimicrobial resistance in general population: A systematic review

**DOI:** 10.1371/journal.pone.0227973

**Published:** 2020-01-16

**Authors:** Hathairat Kosiyaporn, Sunicha Chanvatik, Tibet Issaramalai, Wanwisa Kaewkhankhaeng, Anond Kulthanmanusorn, Nithiwat Saengruang, Woranan Witthayapipopsakul, Shaheda Viriyathorn, Supapat Kirivan, Watinee Kunpeuk, Rapeepong Suphanchaimat, Angkana Lekagul, Viroj Tangcharoensathien

**Affiliations:** 1 International Health Policy Program, Ministry of Public Health, Nonthaburi, Thailand; 2 Division of Epidemiology, Department of Disease Control, Ministry of Public Health, Nonthaburi, Thailand; Chinese Academy of Medical Sciences and Peking Union Medical College, CHINA

## Abstract

**Background:**

Currently, various tools exist to evaluate knowledge and awareness of antibiotic use and antimicrobial resistance (AMR) and are applied by various organizations. Previous systematic reviews have focused mainly on study findings such as levels of knowledge and AMR awareness. However, the survey procedures and data instruments used ought to be scrutinized as well, since they are important contributors to credible results. This review aims to assess the study methods and procedures of existing population-based surveys and explore key components which determine the general population’s levels of knowledge and awareness of antibiotic use and AMR.

**Methods:**

We searched existing literature for population -based surveys which sought knowledge and awareness of antibiotic use or AMR in the general population. Databases searched included Ovid, MEDLINE and EMBASE, PsycINFO and Scopus, domestic journals and gray literature sources. Population-based cross-sectional studies published in English or Thai from January 2000 to December 2018 were included in the review. Quality assessment was conducted using the ‘Appraisal Tool for Cross-Sectional Studies’ (AXIS).

**Results:**

All 22 studies included in the analysis had clear objectives focusing on assessing people’s levels of knowledge, awareness, attitudes and behavior relating to antibiotic use and awareness of AMR. These studies had employed appropriate methodologies for population-based cross-sectional surveys relative to research questions. More than half of studies (14 out of 22) had scientifically soundly designed methodologies which captured the representativeness of the population; whereas the remaining studies had unclear sample size estimations, inappropriate sample frames and selection biases. Half of the studies had tested the validity and reliability of the questionnaire. The common questions used by these surveys were categorized into four themes: behavior related to antibiotic use, knowledge and awareness of antibiotic use, knowledge and awareness of AMR and others such as receiving information about antibiotic use and AMR or cross-cutting issues like self-medication.

**Conclusion:**

This review identified four key features of good practices in antibiotic use and awareness surveys: a) clear survey objective; b) scientifically sound sampling techniques ensuring representativeness; c) strategies for recruitment of samples and survey administration methods; and d) credible measurement to prevent non-sampling biases. During questionnaire design, the health systems context in terms of access to health services and antibiotics should be taken into account. In conclusion, to maximize the use of surveys, the application of findings in surveys and associated factors related to antibiotic use and AMR should primarily generate public health interventions and target specific groups to make progress in solving AMR problems.

## Introduction

Global efforts to assess public knowledge and awareness of antibiotic use and antimicrobial resistance (AMR) are underway. In 2015, the World Health Organization (WHO) developed a questionnaire survey to assess current public knowledge and awareness and behaviors related to antibiotic use in six WHO regions [1]. Similarly, multi-country surveys exist in Europe which use a common protocol, questionnaire and interview methodology [2,3,4]. These population-based surveys are part of the monitoring and evaluation framework proposed by the WHO Global Action Plan on Antimicrobial Resistance (AMR).

In recent years the quest to halt AMR has been materialized in many countries. Thailand is amongst the exemplary countries that made substantial effort to counter AMR. One of the five goals of Thailand’s National Strategic Plan on Antimicrobial Resistance (2017–2021) is to increase public knowledge and awareness of antibiotic use and AMR by 20% before 2021 [5]. The Thai working group on Health Systems and Policy Research on AMR has developed an AMR module and embedded it into the existing biennial Health Welfare Survey (HWS) conducted by National Statistical Office. The aims are to assess among Thai adults the volume of antibiotic use, levels of knowledge on antibiotic use and AMR, exposure to information related to antibiotic use and AMR, and awareness of the use of antibiotics in farm animals. The HWS in 2017 has provided a baseline level of knowledge and AMR awareness in adult populations as required by the National Strategic Plan for monitoring progress against the target [6].

Embedding an AMR module in national surveys has various advantages, such as the possibility for long-term monitoring and opportunities to assess factors associated with knowledge and awareness; it also saves costs compared with conducting independent surveys. Moreover, the merit of survey provides better understanding on behavioral pattern on antibiotic use (either misuse or overuse) in the population, which is one of the key contributing factors to the emergence of AMR. Survey information can serve as a basis to demonstrate an association between knowledge/awareness/practices on antibiotic use and AMR. Thus, it is necessary to establish suitable antibiotic use surveys with associated factors such as knowledge and awareness, in order to tackle with the rising of AMR trends.

However, the national survey on AMR almost always face some difficulties and limitations such as difficulties in the analysis to claim causal relationships, the presence of limited number of independent parameters and information bias when respondents do not understand the questions. This module was adapted from international survey tools such as the Eurobarometer survey in 2009, 2013 and 2016 [2,3,4] and the WHO tool [1], which also present challenges around generalizability and measurement bias, especially in different country contexts. Also, recently published systematic reviews relating to knowledge and awareness of antibiotic use and AMR focused on the results of the surveys [7,8] rather than presenting a review and recommendation of the survey methods and tools, which ultimately influenced the credibility of results.

In attempts to fill existing knowledge gaps pertaining to survey instruments, this review aims to assess the procedures of population-based surveys that ensures representativeness and minimizes biases. It also explores common contents in the questions used by these population-based surveys and categorizes them into thematic areas. The review findings are useful for countries seeking to develop methods and tools to monitor population knowledge and awareness of antibiotic use and AMR in response to the Global Action Plan on AMR.

## Material and methods

This review was registered with PROSPERO database (CRD42019123385) to review protocol: search strategy, inclusion and exclusion criteria, quality assessment and data extraction.

### Search strategy

Search terms were developed along three domains: a) antibiotics or antimicrobial resistance; b) knowledge or awareness; and c) survey or questionnaire. Four international databases (Ovid MEDLINE, Ovid EMBASE, PsycINFO and Scopus) were searched using the search terms as detailed in Table 1. The search terms for international publications were applied to title, abstract, keyword, and full text. Three domestic journals (Health System Research Institute Journal, Thai Journal Citation Index Center, and Thai Journal Online) and gray literatures were manually searched.

**Table 1:** Search terms.

Database	Search term				
	Antibiotics/antimicrobial resistance		Knowledge/awareness		Survey/questionnaire
Ovid MEDLINE/ Ovid Embase/ PsycINFO	"antibiotic*".m_titl. OR "anti-bacter*".m_titl. OR "antibacter*".m_titl. OR "antimicrobe*".m_titl. OR "antibacterial drug* ".m_titl. OR "anti-bacterial drug* ".m_titl. OR "antimicrobial drug* ".m_titl. OR "antibacterial agent* ".m_titl. OR "anti-bacterial agent* ".m_titl. OR "antimicrobial agent* ".m_titl. OR (antibiotic* adj3 resistan*).m_titl. OR (anti-bacter* adj3 resistan*).m_titl. OR (antibacter* adj3 resistan*).m_titl. OR (antimicrob* adj3 resistan*).m_titl. OR ("bacterial drug*" adj3 resistan*).m_titl. OR ("microbial drug*" adj3 resistan*).m_titl. OR Anti-Bacterial Agents/ or Drug Resistance, Bacterial/) OR (antibiotic* adj3 us*).m_titl. OR (antibiotic* adj3 misuse*).m_titl. OR (antibiotic* adj3 overuse*).m_titl. OR "self-medicat*".m_titl. OR Self Medication/	AND	"knowledge*".m_titl. OR "understand*".m_titl. OR "aware*".m_titl. OR "perception*".m_titl. OR "perceiv*".m_titl. OR "attitud*".m_titl. OR "view*".m_titl. OR "opinion*".m_titl. OR "belie*".m_titl. OR "concern*".m_titl. OR "fear*".m_titl. OR "accept*".m_titl. OR "perspectiv*".m_titl. OR "worr*".m_titl. OR "concept*".m_titl. OR KNOWLEDGE/ or HEALTH KNOWLEDGE, ATTITUDES, PRACTICE/ or PATIENT MEDICATION KNOWLEDGE/ OR PERCEPTION/ or SOCIAL PERCEPTION/ OR Attitude to Health/ OR Attitude/ or PUBLIC OPINION/ or "Surveys and Questionnaires"/ OR FEAR/	AND	"assess*".m_titl. OR "evaluat*".m_titl. OR "determin*".m_titl. OR "explor*".m_titl. OR "apprais*".m_titl. OR "estimat*".m_titl. OR "analy*".m_titl. OR "examin*".m_titl. OR "measure*".m_titl. OR "survey*".m_titl. OR "questionnaire*".m_titl. OR "inspect*".m_titl. OR "Surveys and Questionnaires"/
Scopus	TITLE-ABS (antibiotic* OR anti-bacter* OR antibacter* OR antimicrob* OR {antibacterial drug*} OR {antimicrobial drug*} OR {antibacterial agent*} OR {anti-bacterial agent*} OR {antimicrobial agent*} OR anti-bacter* W/3 resistan* OR antibiotic* W/3 resistan* OR antimicrob* W/3 resistan* OR {bacterial drug*} W/3 resistan* OR {microbial drug*} W/3 resistan* OR antibiotic* W/3 usage* OR antibiotic* W/3 misuse* OR antibiotic* W/3 overuse* OR self-medicat*)	AND	TITLE-ABS (knowledge* OR aware* OR understand* OR attitude* OR view* OR perception* OR perceiv* OR opinion* OR belie* OR concern* OR fear* OR accept* OR perspectiv* OR worr* OR concept*)	AND	TITLE-ABS (evaluat* OR determin* OR explor* OR apprais* OR estimat* OR analy* OR examin* OR measure* OR survey* OR questionnaire* OR inspect*)
	(EXCLUDE (PUBYEAR, 1999) OR EXCLUDE (PUBYEAR, 1998) OR EXCLUDE (PUBYEAR, 1997) OR EXCLUDE (PUBYEAR, 1993)) AND (LIMIT-TO (LANGUAGE, "English"))				

### Eligible criteria

The inclusion criteria comprised publications on population-based cross-sectional surveys in the general population which had investigated either knowledge or awareness of antibiotic use or AMR. Publications in English or Thai from international and domestic peer reviewed journals, and gray literature sources which were published between January 2000 and December 2018 were included. Studies on specific population groups, clinical research or studies which could not be electronically retrieved were excluded from the review.

### Study selection

Four researchers (HK, SC, TI and WK) were responsible for abstract screening and full paper review for eligibility. Two researchers screened titles and abstracts to see if they met eligible criteria. The abstracts were included by a consensus between the two researchers and a third opinion was sought if they disagreed. The same process was conducted for the full paper review to select the papers related to research question in term of household-based surveys.

### Quality assessment

The quality of the eligible publications was assessed using the “Appraisal Tool for Cross-Sectional Studies (AXIS)”. AXIS is a descriptive quality assessment tool designed for critical assessment of cross-sectional surveys [9,10]. Using AXIS, the studies were appraised based on five main components: objective, methods, results, discussion and ethics and funding. At this stage, nine researchers (five new—AK, NS, WW, SV, SK—and the four who worked in the study selection process) were grouped into three teams of two or three members to assess the full texts. If there was a disagreement among members of each team, the principal investigator (HK) was responsible for making a final decision.

## Data extraction

Data extraction was conducted into three sets: a) characteristics of studies: author, year of publication, objective, country, study design, sample size, eligible criteria, administration and tool development; b) themes emerging from common questions asked in the surveys to determine level of knowledge and awareness of antibiotic use and AMR or any relevant issues; and c) key findings in the studies.

## Results

An electronic search comprising the four international databases and hand search of three domestic databases and international and domestic gray literature sources yielded a total of 2,761 records (2,740 from the databases and 21 from other sources). After duplicate removal, there were 2,663 papers for abstract screening. 2,537 papers were excluded as they were not relevant leaving 120 records to be searched for full texts. Thirteen full papers that were not electronically available and thirteen duplicates were excluded. 94 full papers were reviewed for eligibility and 72 publications were excluded for not being relevant or pertinent to the review objectives. Finally, 22 studies met the eligible criteria and were included for analysis.

**Fig 1:**
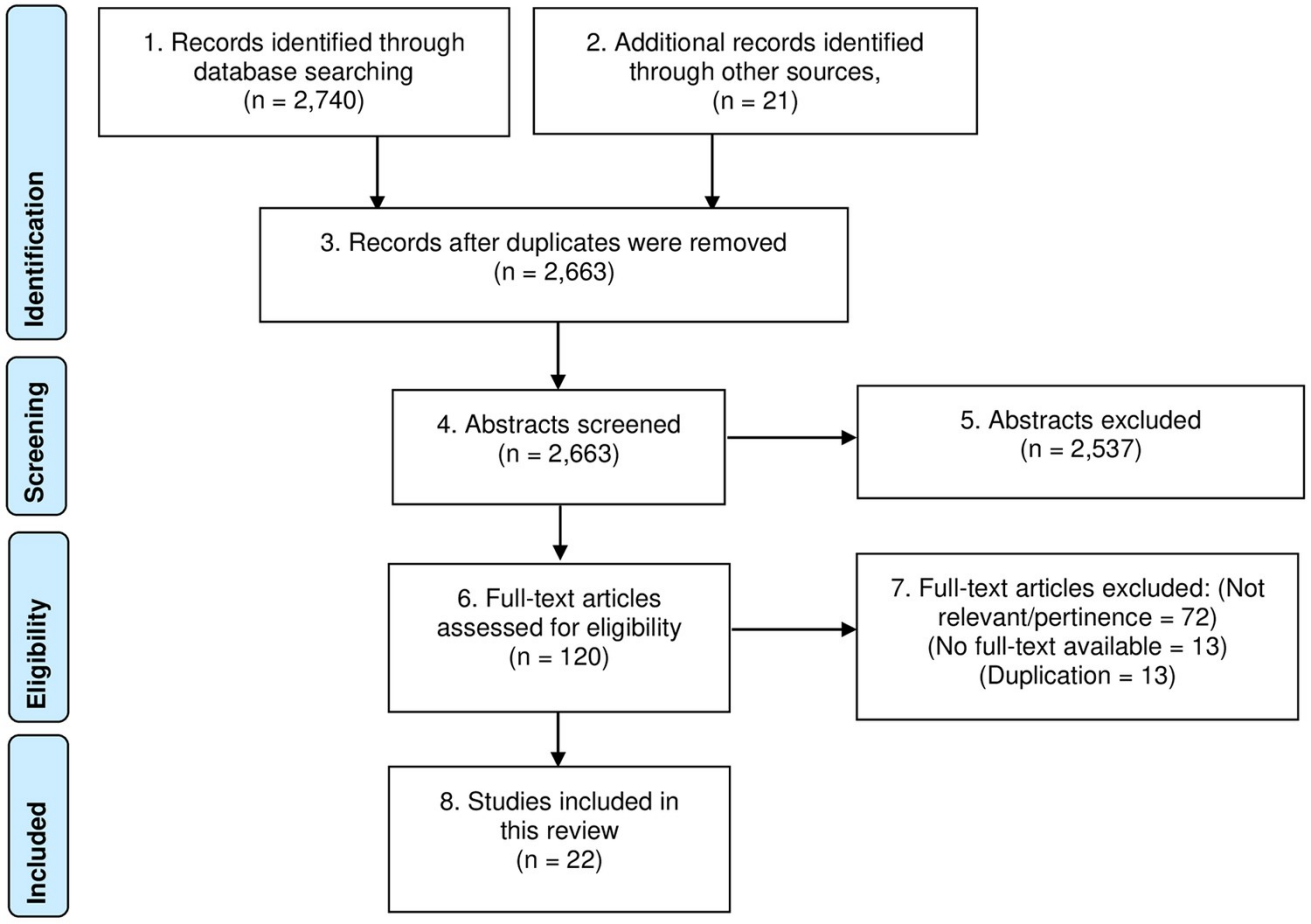
PRISMA flow of systematic review of the survey tools for determining level of knowledge and awareness of antibiotic use and antimicrobial resistance in general population.

A PRISMA flow describing the study recruitment process of this systematic review is shown in Fig 1.

### Characteristic of 22 studies

Almost all (19 studies) were published articles in peer-reviewed journals while 3 were reports [1,5,11]. Of 22 studies, 20 were published in international peer-reviewed journals while 2 published in domestic journals [6,11].

Table 2 summarizes characteristics of the 22 studies. There were 19 out of 22 papers published from 2010 to 2018.

**Table 2:** Characteristics of 22 included studies.

No	Author	Year of publication	Objective of study	Study design	Country	Inclusion criteria	Exclusion criteria	Number of respondents/ Sample size	Sampling technique	Administration	Reference of tool development	Key findings
1	Parimi N. et al.	2002	To determine the general public’s perceptions and use of antibiotics in Trinidad and Tobago, a two-island republic in the Caribbean	Observational study	Trinidad and Tobago	Household members who were at least 18 years old and take care of family members when they were ill	NA	753/800	Random sampling	Telephone surveys	NA	93% of the respondents knew the term “antibiotic”. Penicillin was correctly identified as common antibiotic but 36% of the respondents incorrectly identified Benadryl (diphenhydramine), a common over-the-counter drug for cough and cold formulation, was antibiotic. Beta-lactams were the most frequently used antibiotics in the previous year, and 20% of antibiotics users used multiple antibiotics. A quarter of the respondents had asked a doctor for antibiotic prescriptions. 29% of the respondents said that antibiotics are drugs for treating bacterial infections. Respondents who have completed tertiary education (university) was significantly associated with correct knowledge of the safety of antibiotics. Respondents, who had private health insurance, were more likely to say that antibiotics are safe and do not have side effects, and incorrectly classified aspirin and Benadryl as antibiotics compared to those without private health insurance.
2	Eng JV. et al.	2003	To provide a glimpse of the current knowledge, attitudes, and practices regarding antibiotic use among patients	Observational study	Connecticut, Minnesota, Oregon, and selected counties in California, Georgia, Maryland and New York, USA	Household members	NA	12,755/NA	Single-stage random sampling	Telephone surveys	NA	12% of the respondents had recently taken antibiotics in the past 4 weeks. 27% of the respondents believed that taking antibiotics when they suffered from a common cold made them feel better more quickly. 32% of the respondents believed that taking antibiotics helped preventing more serious illnesses. 48% of the respondents expected a prescription for antibiotics when they were ill. 58% of respondents were not aware of health dangers associated with taking antibiotics.
3	McNulty CAM. et al.	2007	To assess the respondents’ knowledge and attitudes to antibiotics, their reported antibiotic use and the relationship with household and respondent characteristics, and investigate what sort of person was more likely to be aware of the Antibiotic campaign	Interventional study	Great Britain (England, Wales and Scotland)	Household members who aged over 16 years old	NA	7,120/10,981	Stratified random sampling with proportional to size	Face-to-face interviews	NA	About 40% of the respondents neither knew that antibiotics do not work against most coughs or colds nor knew that antibiotics. can kill normal flora on skin and in the gut. 79% of the respondents were aware of antibiotic resistance in British hospitals. Respondents with lower level of education were less knowledgeable about antibiotics. Knowledge was positively associated self-seeking of antibiotics. Better knowledge of antibiotics did not always lead to lower antibiotic prescription, but was associated with the completion of a course of antibiotic prescribed.***
4	Andre´ M. et al.	2010	To examine the level of knowledge about antibiotic treatment and awareness of antibiotic resistance among the general public in Sweden	Observational study	Sweden	Aged 21–80 years old	NA	747/1,000	Random sampling	Telephone surveys	NA	19% of the respondents agreed that antibiotics cure common colds more quickly; but most respondents believed that bacteria can become resistant to antibiotics. The respondents showed some confusion over the terms ‘bacteria’ and ‘viruses’, and this confusion partly influences the decision to take antibiotics.
5	Barah F. and Goncalves V.	2010	To provide an insight of the current knowledge and practices regarding antibiotic use among individuals living in the Syrian Arab Republic	Observational study	Kalamoon, Syrian Arab republic	household members	Aged under 18 years old and unable to understand Arabic language	445/556	Random sampling	Face-to-face interviews	Eng JV. et al. (2003)**	85% of the respondents had taken antibiotics in the past 4 weeks; mostly from physician (43%). 57% of the respondents used leftover antibiotics or took someone else’s advice. 34% of the respondents were not aware of the dangers of antibiotics. Males, younger age, and those with low and medium income and lower level of education showed poorer practice and lower level of knowledge on antibiotics and awareness of the health dangers associated with antibiotics.
6	Kim SS. et al.	2011	To examine public level of knowledge and attitudes regarding antibiotic use and potential drug resistance in South Korea	Observational study	Gangwon-do, Seoul, Busan, Daegu, Incheon, Daejeon, Gwangju, South Korea	aged over 18 years old	NA	1,177/1,500	random sampling with proportional to size	face-to-face interviews	USCDC (2010)	70% of the respondents did not know that antibiotics are not effective in treating cough and cold. Two thirds of the respondents were unaware of the conditions under which antibiotic resistance could occur. Lower level of education and older age were significantly associated with inadequate knowledge of antibiotics. Lower level of education, older age, inadequate knowledge and absence of exposure to antibiotic safety campaign were significantly associated with poor attitude towards antibiotics.***
7	Sirijoti K.	2012	To describe the socio-demographic characteristics and assess the level of knowledge, attitude and practice regarding antibiotic use among adults in Kuanthani subdistrict Kantang district, Trang, Thailand	Observational study	Kuanthani Subdistrict, Kantang District, Trang, Thailand	Aged 18 years old and above People who were living in Kuanthani Subdistrict, Kantang District, Trang, Thailand for more than 6 months People who could listen, speak, read and write in Thai language People who were willing to participate in the study	People who were working as health professionals People who were incapable of responding to survey questions because of psychiatric or neurological disorder People who were not available at the time of survey People who were temporary in the city for vacation	396/396	Systematic random sampling with proportional to size	Face-to-face interviews	Eng JV. et al. (2003)**, Buke et al. (2003), Hsiao et al. (2006), Darmanin Ellul et al. (2008), You et al. (2008), Panagakou et al. (2009, 2011), Leochico et al. (2010), Oh et al. (2011), Rousounidis et al. (2011), Shehadeh et al. (2011), Kunnee (1995), Sirirassamee (1997), Na Nakorn (2002), Suwan (2006), Suksomsin (2008), Kaenjan (2008) and Kaewmang (2010), Kaliyaperumal (2004) and WHO (2008)	Mean score of knowledge was 10.43±2.84 (min = 3, max = 16). Mean attitude score was 2.49±0.39 (min = 1.27, max = 3). Mean practice score was 2.68±0.22 (min = 1.81, max = 3). Female, younger age, being single, high education levels and high income were significantly associated with better knowledge and attitudes. There was significant association between knowledge and attitudes, and practices regarding antibiotic use.
8	Widayati A. et al.	2012	To describe knowledge and beliefs about antibiotic use among people in an urban area of Indonesia	Observational study	Yogyakarta, Indonesia	Aged over 18 years old	NA	559/640	Cluster random sampling	Self-administration	Sawair FA. et al. (2009)**, Chetley A. et al. (2007), Panagakou SG. et al. (2009), Sahoo KC. (2008) and Hadi U. et al. (2008)	85% of the respondents had appropriate knowledge regarding antibiotic resistance; 70% had appropriate knowledge about allergic reactions and 76% had appropriate knowledge about antibiotics’ effectiveness for bacterial infections. Half of the respondents knew that antibiotics ought not to be used immediately when catching fever. 71% of the respondents had incorrect knowledge regarding antibiotic benefit for viral infections. 24% of the respondents believed that antibiotics had no side effects. There was a positive association between knowledge and beliefs especially in those who are male, younger age, have higher level of education, and higher income.
9	Wun YT. et al.	2012	To examine the public’s perspectives on antibiotic resistance in our study of the public’s knowledge, attitude and practice with antibiotics in Hong Kong	Observational study (mixed method)	Hong Kong	Household members aged 18 years old or above who are Hong Kong residents speaking local dialects	Persons with communication difficulties	2,471/2,401	Random sampling	Telephone surveys	NA	9% of the respondents had never heard the term ‘antibiotic resistance’. About 8% of the respondents had ever acquired non-prescribed antibiotics. About 7% of the respondents had ever kept the leftover antibiotics while around 70% of the respondents had always finished the full course of antibiotics. About 75–77% of the respondents agreed that the purchase of antibiotics without prescription and incomplete courses of antibiotics will lead to undesirable consequences. About 39% of the respondents agreed that they could help the prevention of resistance.
10	Ahmad H. et al.	2013	To address the attitude, knowledge and perception of Peshawar and Mardan inhabitants towards self-medication	Observational study	Peshawar and Mardan, Pakistan	NA	NA	500/NA	Random sampling	Self-administration	NA	78% of the respondents had used antibiotics without prescriptions. About 26% of the respondents never checked the expiry date on antibiotics they used for self-medication. About 64% of the respondents believed that they knew the indication of antibiotics taken; however, only 34% of the respondents reported that antibiotics can be used to treat dengue fever.
11	Jose J. et al.	2013	To assess public knowledge, belief and behavior of antibiotic use in two representative governorates out of the ten governorates in Oman	Observational study	Al Batnah and Al Dakhliyah governorates, Oman	Members of the public aged between 18–60 years old who understand the term antibiotic and had used an antibiotic at least three times in their lifetime	Healthcare professionals or students from any medical/health related field	718/600	Quota random and convenience sampling	Self-administration and face-to-face interviews	McNulty CAM. et al. (2007)**, Pechère JC. et al. (2007), Kandakai TL. et al. (1996) and Crury M. et al. (2006)	Moderate knowledge and behavior score were observed, while the belief score of the respondents was low. A significant difference was observed in the median total score in respondents from different age groups, education levels and employment status.
12	Gu J. et al.	2015	To explore the differences in the knowledge of, attitude towards and use of antibiotics between urban and rural populations in the Heilongjiang Province of China and review the factors that were associated with the knowledge of, attitude towards and use of antibiotics in this population	Observational study	Suihua, Yichun (Tieli City County area) and Harbin regions of Heilongjiang Province, China	Aged over 18 years old	NA	3,631/NA	Random sampling	Self-administration or with assistance of investigators	NA	More than 60% of the respondents were aware that antibiotics can be used to treat bacterial infections and that bacteria can be resistant to antibiotics. About 40–60% of the respondents were aware that antibiotic resistance had become a major problem in China. Urban participants reported greater level of knowledge of and attitude towards use of antibiotics than rural participants. Logistic regression indicated that urban residency, female and education levels were associated with knowledge of, attitudes towards and use of antibiotics.***
13	Mouhieddine TH. et al.	2015	To assess the current knowledge, attitudes and practices, regarding antibiotic usage in a Lebanese sample and identify demographic characteristics associated with the highest risk of attaining resistance	Observational study	Beirut, Lebanon	Aged at least 18 years old Lived in Lebanon for at least the past 5 years to ensure that they have adapted to Lebanese habits that affect the knowledge, attitude and practice of antibiotic consumption Aware of the term ‘antibiotics’ or any of its marketed equivalents	Respondents who did not meet all criteria	495/500	Random and convenience sampling	Self-administration	Andre´ M. et al. (2010)**, Kim SS. et al. (2011)** and Linh OA. et al. (2011)**	68% of the respondents used antibiotics 1–3 times per year. About 80% of the respondents considered antibiotics as anti-bacterial agents while 74% of the respondents did not know that antibiotics are not anti-viral agents. Approximately 67% of the respondents realized that abusing antibiotics can lead to resistance. Income, education levels, place of residence, having health insurance, history of working in the health sectors and spending a year outside Lebanon were significantly associated with better knowledge and attitude towards antibiotics.
14	WHO	2015	To provide a snapshot of current public awareness and common behaviors related to antibiotics in a range of countries	Observational study	12 countries from all six WHO regions*	Aged over 16 years old	NA	9,772/9,772	Quota random sampling	Face-to-face interviews or online surveys	NA	35% of the respondents reported having taken antibiotics within the past month; mostly from drug stores (93%), and physicians or nurses (81%). 64% of the respondents incorrectly believed that viruses such as colds and flu can be treated with antibiotics. 87% agreeing that people should use antibiotics only when prescribed. 32% of the respondents think that they should stop taking antibiotics when they feel better. 72% of the respondents correctly believed that many infections are becoming increasingly resistant to treatment by antibiotics.
15	Al-Naggar RA. et al.	2016	To examine the level of knowledge, attitude and the associated factors of antibiotic use among urban community in Malaysia	Observational study	SubangBestari, Shah Alam, Selangor, Malaysia	Residents of SubangBestari aged over 18 years old able to read and understand Malay language	NA	450/450	Random sampling	NA	Based on literatures (no references)	About 79% of the respondents reported that antibiotics used to treat bacterial infection while about 53% of those reported that antibiotics used to treat viral infections. About 62% of the respondents were aware of antibiotic resistance in relation to the overuse of antibiotics. About 35% of the respondents reported that when they got cold, they would take antibiotics to help them get better more quickly. Education levels, healthcare-related occupation and family’s occupation related to healthcare were significantly associated with knowledge of antibiotics. Healthcare-related occupation, marital status and income were significantly associated with better attitude. Knowledge score was positively associated with attitude score.
16	European Commission	2016	To identify the use of antibiotics among the EU public To measure the levels of public knowledge about the nature and effectiveness of antibiotics and the risks associated with their unnecessary use To determine the impact of the information Europeans have received To obtain perceptions of the most appropriate policy response to antibiotic resistance; To assess knowledge of and attitudes towards the use of antibiotics in agriculture and the environment	Observational study	28 EU member states	Aged over 15 years old	NA	27,969/28,000	Multi-stage random sampling with proportional to size	Face-to-face interviews	NA	34% of the respondents said that they took antibiotics within the previous year; mostly from health care providers (93%) and for conditions such as bronchitis (18%), flu (16%) and sore throat (14%). 43% of the respondents knew that antibiotics are ineffective against viruses and 56% of those knew that antibiotics are also ineffective against colds and flu. 84% of the respondents knew that unnecessary use of antibiotics makes them become ineffective. 82% of the respondents think they should stop taking antibiotics once they begun a course of treatment. 33% of the respondents remembered that they received information about the unnecessary use of antibiotics in the last 12 months; mostly from health professionals.
17	Vallin M. et al.	2016	To provide an update on the knowledge and attitudes to antibiotic use and resistance of the Swedish population and identify which groups within the population are in particular need of improved knowledge or attitudes	Observational study	Sweden	Aged between 18 and 74 years old who lived in Sweden including Swedish and foreign citizens	NA	1,426/2,500	Random sampling	Mail	Andre´ M. et al. (2010)**	94% of the respondents knew that bacteria could become resistant to antibiotics. Male, younger age and educated people were more likely to be knowledgeable but male had a less restrictive attitude The respondents with high level of knowledge on antibiotics were more likely to have appropriate restrictive attitudes towards antibiotics.***
18	Mazińska B. et al.	2017	To assess knowledge by the general public in Poland regarding antibiotics, AMR, and the impact of the European Antibiotic Awareness Day campaigns	Interventional study	Poland	Aged over 18 years	NA	5,004/5,000	Multi-stage and stratified random sampling	Telephone surveys	Eurobarometer Survey 338 (2014)**	38% of the respondents had used antibiotics within the past 12 months; mostly from physicians (90%) About 40% of the respondents expected a prescription for an antibiotic against flu. 80% of the respondents knew that antibiotics kill bacteria while 60% of those believed antibiotics kill viruses. 29% of the respondents declared to have come across information on the prudent use of antibiotics in the preceding 12 months and 48% of those declared that the information resulted in the change of attitude towards antibiotic use.
19	Zajmi D. et al.	2017	To assess the level of knowledge, attitudes and practices about antibiotic use among the general public in Kosovo	Observational study	Kosovo	Aged over 15 years old	NA	811/770	Stratified random sampling with proportional to size	Face-to-face interviews	Special Eurobarometer 407 (2013)**	About 59% of the respondents used antibiotics within the previous year, mostly for conditions such as flu (24%), sore throat (20%), cold (13%) and common cold (8%). About 43% of the respondents opined that antibiotics are effective against viral infections. 47% of the respondents received information about the unnecessary use of antibiotics and 33% of those reported that it changed their views and behaviors after receiving the information.
20	Chanvatik S. et al.	2018	To better understand the appropriate use of antibiotics and monitor as well as evaluate of implementing the National Strategic Plan on Antimicrobial Resistance 2017–2021	Observational study	Thailand	Aged over 15 years old who response to questionnaires by themselves	NA	27,762/27,960	Stratified two-stage random sampling	Face-to-face interviews	Special Eurobarometer 445 (2016)**	About 8% of the respondents received antimicrobial drugs in the last month; mostly for respiratory symptoms (63%) and health facilities (70%). About 3% of the respondents showed correct answers to all statements and most incorrect answers were “antimicrobials can kill viruses” and “antimicrobials are effective against colds and flu”. About 18% of the respondents received information about proper use of antimicrobials in the last 12 months; mostly from health professionals.
21	Haenssgen MJ. et al.	2018	To inform the awareness agenda from a social sciences perspective by assessing the outputs, outcomes, and behavioral impacts of an antibiotic resistance-themed educational activity in the low-income setting of Southern Lao PDR	Interventional study	Salavan, Lao PDR	aged over 18 year who were Laos villagers and lived in this area more than six months	adolescents and children, people who unable to participate in the study after two attempts to arrange interview	2,480 (1264 in round I and 1216 in round II)/2,480	consensus	face-to-face interviews	Haenssgen,MJ et al. (2018)**	Activity-related educational activities could positively influence the awareness and understanding of “drug resistance”, whereas its effects on attitudes were minor. The evidence on the behavioral impacts was sparse and mixed. One of the possible influences included a disproportionate uptake of antibiotics from formal healthcare providers.
22	Salm F. et al.	2018	To investigate the history of antibiotic use in the general population and to characterize consumers in terms of health literacy and knowledge	Observational study	Berlin, Germany	Aged over 35 years old Sufficient German language skills Resident of Germany	No	977/2000	Stratified random sampling	Face-to-face interviews	WHO (2015)** and Gualano et al. (2015)	About 33% of the respondents indicated having had an antimicrobial prescription during the previous 12 months. Individuals with sufficient health literacy were only 0.57 times less likely to have had a recent history of antibiotic use than individuals with insufficient health literacy.***

Note:

* Barbados, China, Egypt, India, Indonesia, Mexico, Nigeria, Russian Federation, Serbia, South Africa, Sudan, Vietnam

** Meet inclusion criteria of this paper

***Multivariate analysis, all data about association was significant at p-value<0.05

The majority of the studies (20 studies) were conducted as single-country studies while 2 studies were multi-country studies at global and regional levels [1,4]. Among the 20 single-country studies, 8 studies were conducted at national level [6,12,13,14,15,16,17,18] while the remaining 12 studies were conducted at sub-national level [11,19,20,21,22,23,24,25,26,27,28,29]. It should be noted that no study was conducted in the African region.

Regarding study design, 3 out of 22 studies were interventional studies which assessed the outcomes of campaign and educational interventions on the proper use of antibiotics [13,17,28]. The remaining 19 publications were observational studies. Only one study [15] was conducted by using mixed methods while the other 21 studies applied quantitative methods.

Sample size varied depending on the sampling frame and approaches employed by each study. More than half of the studies [1,4,6,11,12,13,15,17,18,21,24,27,29] mentioned that sample size was calculated based on statistical method and population data. Sample size varied from less than 400 [11] to more than 27,000 individuals [4,6].

For sampling criteria, nearly half of studies (10 out of 22) recruited only adults over 15, 18 or 21 years old [1,4,6,13,14,17,18,21,22,25]. Some studies [11,12,15,16,24,26,27,28,29] had additional criteria such as respondents’ understanding of local languages, familiarity with the term “antibiotics” or whether they had lived in households or the geographical area for a certain period. Almost all studies (21 studies) described specific administration methods [1,4,6,11,12,13,14,15,16,17,18,19,20,21,22,23,24,25,26,28,29]. Nine out of twenty-one applied only randomized sampling techniques with face-to-face interviews using a structured interview questionnaire [4,6,11,13,18,20,21,28,29]. Other administration methods were less common, such as telephone interview surveys, and self-administered questionnaire surveys using mail and online channels [1,12,14,15,16,17,19,22,23,24,25,26].

### Quality assessment of 22 studies

The results of the quality assessment of 22 eligible studies by using the AXIS tool are shown in Tables 3 and 4.

**Table 3:** Quality assessment of 22 included studies using Appraisal Tool for Cross-Sectional Studies (AXIS).

	Introduction	Methods									
Author (Year of publication)	Were the aims/objectives of the study clear?	Was the study design appropriate for the stated aim(s)?	Was the sample size justified?	Was the target/ reference population clearly defined? (Is it clear who the research was about?)	Was the sample frame taken from an appropriate population base so that it closely represented the target/reference population under investigation?	Was the selection process likely to select subjects/participants that were representative of the target/ reference population under investigation?	Were measures undertaken to address and categorize non-responders?	Were the risk factors and outcome variables measured appropriate to the aims of the study?	Were the risk factors and outcome variables measured correctly using instruments/ measurements that had been trialed, piloted or published previously?	Is it clear what was used to determined statistical significance and/or precision estimates? (e.g., p values,CIs)	Were the methods (including statistical methods) sufficiently described to enable them to be repeated?
Parimi N. et al (2002)	Y	Y	Y	Y	Y	N	N	Y	Y	Y	Y
Eng JV. et al. (2003)	Y	Y	N	Y	Y	Y	N	N	N	Y	Y
McNulty CAM. et al. (2007)	Y	Y	Y	Y	Y	Y	N	N	N	Y	Y
Andre´ M. et al (2010)	Y	Y	N	Y	Y	Y	N	N	Y	Y	Y
Barah F. and Goncalves V. (2010)	Y	Y	N	Y	N	Y	N	CT	Y	Y	Y
Kim SS. et al (2011)	Y	Y	Y	Y	Y	Y	N	Y	Y	Y	Y
Sirijoti K. (2012)	Y	Y	Y	Y	Y	Y	N	Y	Y	Y	Y
Widayati A. et al (2012)	Y	Y	N	Y	Y	Y	CT***	Y	Y	Y	Y
Wun YT. et al. (2012)	Y	Y	Y	Y	Y	Y	N	Y	Y	Y	Y
Ahmad H. et al (2013)	Y	Y	N	Y	Y	N	N	N	N	N	N
Jose J. et al (2013)	Y	Y	Y	Y	Y	N	N	Y	Y	Y	Y
Gu J. et al (2015)	Y	Y	N	Y	Y	Y	N	N	N	Y	Y
Mouhieddine HT. et al (2015)	Y	Y	N	Y	N	N	N	Y	Y	Y	Y
WHO (2015)	Y	Y	Y	Y	Y	Y	N	N	N	N	Y
Al-Naggar AR. et al (2016)	Y	Y	Y	Y	Y	Y	N	Y	Y	Y	Y
European Commission (2016)	Y	Y	Y	Y	Y	Y	N	N	N	Y	Y
Vallin M. et al (2016)	Y	Y	N	Y	Y	Y	Y	Y	Y	Y	Y
Mazińska B. et al (2017)	Y	Y	Y	Y	Y	Y	N	Y	N	Y	Y
Zajmi D. et al (2017)	Y	Y	Y	Y	Y	Y	N	Y	Y	Y	Y
Chanvatik S. et al (2018)	Y	Y	Y	Y	Y	Y	N	N	N	Y	Y
Haenssgen JM. et al (2018)	Y	Y	Y	Y	Y	Y	N	N	N	Y	Y
Salm F. et al (2018)	Y	Y	Y	Y	Y	N	N	Y	Y	Y	Y

Note: Y = Yes, N = No, CT = Cannot Tell

**Table 4:** Quality assessment of 22 included studies using Appraisal Tool for Cross-Sectional Studies (AXIS).

	Results					Discussion		Others	
Author (Year of publication)	Were the basic data adequately described?	Does the response rate raise concerns about non-response bias?	If appropriate, was information about non-responders described?	Were the results internally consistent?	Were the results for the analyses described in the methods, presented?	Were the authors’ discussions and conclusions justified by the results?	Were the limitations of the study discussed?	Were there any funding sources or conflicts of interest that may affect the authors’ interpretation of the results?	Was ethical approval or consent of participants attained?
Parimi N. et al (2002)	Y	N	N	Y	Y	Y	Y	CT	Y**
Eng JV. et al. (2003)	Y	CT	CT	Y	Y	Y	Y	N	Y
McNulty CAM. et al. (2007)	Y	N	N	Y	Y	Y	Y	N	N*
Andre´ M. et al (2010)	Y	N	N	Y	Y	Y	Y	N	Y
Barah F. and Goncalves V. (2010)	Y	N	N	Y	Y	N	Y	N	Y
Kim SS. et al (2011)	Y	N	N	Y	Y	Y	Y	N	N*
Sirijoti K. (2012)	Y	N	N	Y	Y	N	Y	N	Y
Widayati A. et al (2012)	Y	CT***	CT***	Y	Y	Y	Y	N	Y
Wun YT. et al. (2012)	Y	N	N	Y	Y	Y	Y	N	Y
Ahmad H. et al (2013)	N	CT	CT	CT	Y	N	N	CT	Y
Jose J. et al (2013)	Y	N	N	Y	Y	Y	Y	N	Y
Gu J. et al (2015)	Y	CT	CT	N	Y	Y	Y	N	Y
Mouhieddine HT. et al (2015)	Y	N	N	Y	Y	N	Y	N	Y
WHO (2015)	Y	N	N	CT	Y	Y	Y	CT	CT
Al-Naggar AR. et al (2016)	Y	N	N	Y	Y	N	N	CT	Y
European Commission (2016)	N	N	N	Y	Y	CT	N	CT	CT
Vallin M. et al (2016)	Y	Y	Y	Y	Y	Y	Y	N	Y
Mazińska B. et al (2017)	N	N	N	Y	Y	Y	N	N	Y
Zajmi D. et al (2017)	Y	N	N	Y	Y	Y	Y	N	Y
Chanvatik S. et al (2018)	Y	N	N	Y	Y	Y	N	N	N*
Haenssgen JM. et al (2018)	Y	N	N	N	Y	Y	Y	N	Y
Salm F. et al (2018)	Y	N	N	Y	Y	N	Y	N	Y

Note: Y = Yes, N = No, CT = Cannot Tell

* Exemption for ethical approval

** Only consent of respondents attained

*** This study did not categorize non-responders but it compared the characteristics of respondents who were familiar and were not non-familiar with antibiotics.

Our analysis found that all publications had clear study objectives which focused on assessing the levels of knowledge and awareness or attitudes and behavior related to antibiotic use and AMR and associated factors.

All studies employed the appropriate methodology of cross-sectional survey related to objectives. There were 14 studies [1,4,6,11,12,13,14,15,17,18,21,24,28,29] that reported an estimation of sample size using justified statistical methods. All studies clearly defined the reference population but two studies [20,26] had inappropriate sample frame and five studies [12,13,14,26,29] had selection process that tended to be non-representative.

Half of the studies [11,12,15,16,18,21,22,24,26,27,29] tested both the validity and reliability of the questionnaire and provided the statistical significance of key variables. Only one study [23] insufficiently described the method which was difficult to repeat.

Regarding the reporting of survey results, almost all studies presented adequate basic data and all results for the described methods, except three studies [1,4,23] which did not show basic data. The results in two studies [25,28] were not internally consistent and one study [1] could not be identified due to showing only percentage data. Vallin M. et al (2016) was the only study which addressed and categorized non-responders and which reported profiles of non-respondents to the survey. Widayati A. et al (2012) did not categorize data of non-responders, but they compared the characteristics of respondents who were familiar and not non-familiar with antibiotics and found no difference between these two groups.

Seventeen studies discussed limitations including selection biases [1,12,14,15,21,22,24,25,26,28], information biases such as recall biases [1,12,13,14,16,18,19,20,21,24,26,28], measurement bias [1,29], study design limitations [11,13,16,20,21,25] and other possible confounders [18,19,29].

Regarding conflict of interests and ethical reviews, five studies did not clearly declare funding sources which might influence authors’ interpretation of the results [1,4,12,23,27]. Seventeen studies indicated they had ethical approval or consent of the participants. Another five studies [1,4,6,12,13] did not provide information on ethical clearance or whether they attained consent of the survey participants. Three studies [6,13,21] declared that their studies were exempted from ethical review.

According to AXIS quality assessment, the tool does not provide a numeric scale for assessment, but it is flexible enough for users to judge the quality of the paper overall. However, authors in this systematic review classified all papers into three types based on methodology, results and discussions: 1) fully qualified; 2) partly qualified; and 3) unqualified. Fully qualified means the studies are qualified in all parts; there are no studies which reach this qualification. Partly qualified considers the studies that have qualifications in some parts; there are six studies in this group [11,15,16,18,21,27]. Four studies [15,16,18,21] are not fully qualified in methodology while the other two studies [11,27] are not fully qualified in discussion. Kim SS. et al (2011), Wun YT. et al. (2012) and Zajmi D. et al (2017) did not mention about non-response biases whereas Vallin M. et al (2016) did not reported calculation method for sample size. Sixteen studies are unqualified because they are missing important parts of quality assessment; for example, some studies had inappropriate selection processes influencing representativeness and some studies did not have validity and reliability tests of measurements.

### Thematic concerns of questions in the questionnaire survey

Of the 22 studies, 13 [6,11,16,17,18,20,21,22,24,26,27,28,29] adapted a questionnaire from prior studies, and the questionnaire for household-based cross-sectional surveys in general population from the Eurobarometer survey (2013,2014,2016), Andre´ M. et al. (2010) and Eng JV. et al. (2003) was commonly referred to.

Four themes emerged from the analysis of the contents of the questionnaire: a) behavior related to antibiotic use; b) knowledge and awareness of antibiotic use; c) knowledge and awareness of AMR and d) other issues such as receiving information and advice about proper use of antibiotics, or AMR campaign message and cross-cutting issues such as self-medication. See Table 5.

**Table 5:** Common questions used to determine level of knowledge and awareness of antibiotic use and AMR.

Themes	Subthemes	Common questions/statements
Behavior related to antibiotic use	Frequency of using antibiotics	Have you taken any antibiotics in the last one month or 12 months?
	Source of antibiotics	How do you obtain the antibiotics?
	Indication/reason of antibiotic use	What was the reason for last taking the antibiotics that you used?
	Instruction of antibiotic use	Do you read the label information medicine name and indication of antibiotics before taking it?, Do you drink alcohol while taking antibiotics?, etc. (Yes/No)
Knowledge and awareness of antibiotic use	Name of antibiotics	Please identify the name of antibiotics e.g. penicillin, tetracycline, etc.
	General knowledge	Antibiotics can kill bacteria. (Yes/No)
		Antibiotics can kill viruses. (Yes/No)
		Antibiotics can treat colds and flu (Yes/No)
		Antibiotics can treat symptoms such as fever, cough, pain and inflammation, etc. (Yes/No)
		Antibiotics have side-effects such as diarrhea, nausea and vomiting (Yes/No)
		People can be allergic to antibiotics (Yes/No)
		Unnecessary use of antibiotics makes them become ineffective (Yes/No)
	Awareness of using antibiotics in common cold/flu	When I have a cold, I should take antibiotics to prevent getting a more serious illness (Agree/Disagree)
		When I get a cold, antibiotics help me to get better more quickly (Agree/Disagree)
		By the time I am sick enough to talk to or visit a doctor because of a cold, I usually expect a prescription for antibiotics (Agree/Disagree)
Knowledge and awareness of AMR	Definition	Antibiotic resistance means that bacteria would not be killed by antibiotics (Yes/No)
	General knowledge	When antibiotics are taken for the wrong indication such as incomplete course or lower doses, it can lead to antibiotic resistance (Yes/No)
		Overuse of antibiotics can cause antibiotic resistance (Yes/No)
		Bacteria which are resistant to antibiotics can be spread from person to person (Yes/No)
	Awareness	Antibiotic resistance is a problem in your country and worldwide (Agree/Disagree)
		Antibiotic resistance is an issue that could affect me or my family (Agree/Disagree)
Others	Information about antibiotic use and AMR	In the last 12 months, do you remember getting any information about antibiotic use or AMR, for example, messages about not taking antibiotics in case of cold or flu? (Yes/No)
		What are the sources of information on antibiotic use or AMR?
		Did information that you received change your views/behaviors on using antibiotics? (Yes/No)
	Self-medication with antibiotics	You can stop taking a full course of antibiotic if your symptoms are improving (Yes/No)
		You can share antibiotics from and to person who have experienced the same symptoms as you (Yes/No)
		You can keep leftover antibiotics and use later in the future (Yes/No)
	Patient-doctor relationship	I trust the doctor’s decision when s/he prescribes antibiotics. (Agree/Disagree and Yes/No)
		Doctors and pharmacists often take time to inform the patient during the consultation about how antibiotics should be used. (Agree/Disagree and Yes/No)

With regard to behavior-related antibiotic use, we identified four sub-themes covering: 1) frequency of using antibiotics in the recall period such as one month, six months or a year; 2) source of antibiotics; 3) clinical indications or conditions for which antibiotics are used; and 4) instruction and advice from drug sellers or pharmacists on the proper use of antibiotics.

For knowledge and awareness of antibiotic use, three sub-themes emerged: 1) antibiotic names; 2) general knowledge; and 3) awareness of using antibiotics in common cold and flu symptoms. Questions were asked about respondents’ recognition of antibiotics, for example whether penicillin or tetracycline were antibiotics or not. In terms of general knowledge, questions were asked about the mechanism of action of antibiotics, such as its action towards bacteria or viruses, its side-effects and allergies, and inappropriate antibiotic use. Finally, concerning awareness of antibiotic use, questions were designed to explore opinions about antibiotic use for common cold and flu symptoms.

On knowledge and awareness of AMR, various questions in the survey tools were categorized into three subthemes: 1) definition of AMR; 2) general knowledge about AMR; and 3) awareness of AMR. The general knowledge questions focused on misuse, overuse, sub-optimal use and inappropriate use of antibiotics, which could lead to AMR and the spread of resistant bacteria. Concerning awareness of AMR, various questions explored people’s concerns about AMR, which had the potential to affect themselves, their families and countries.

Self-medication with antibiotics is a cross-cutting issue in all the three thematic areas. Questions explored the necessity of completing the full course of antibiotics and proper management of the leftover antibiotics.

Additional questions explored exposure to public information relating to proper use of antibiotics and AMR. These included media channels and sources of information such as health professionals, and the impact of this information on people’s behavior in relation to antibiotic use. For doctor-patient relationships, the questions related to trust and communication between people and healthcare providers.

## Discussion

The systematic review observed several important features in design and methodology of included studies that would be useful for developing a tool to determine levels of knowledge and awareness of antibiotic use and AMR.

Setting objectives is vital to guide study design and all included studies had clear objectives focusing on assessing levels of knowledge, awareness or attitudes and behavior related to antibiotic use and awareness of AMR and associated factors. A cross-sectional survey is appropriate for the assessment of population knowledge about and awareness of proper use of antibiotics under the resource constraints. It measures exposure and outcomes at the same time and can find possible associations between exposure and outcomes [30]. Cross-sectional surveys are less costly and less time-consuming than longitudinal studies [30]. However, the casual relationships are better identified through longitudinal studies where temporal relationship can be addressed [31]. A recent systematic review on public knowledge and beliefs about AMR has shown that synthesis of qualitative and quantitative studies provided more in-depth understanding of people’s knowledge and beliefs about AMR than using quantitative data alone [8]. In this review, the number of quantitative studies was three times higher than qualitative studies and mixed methods. Due to the strengths and limitations of each method, quantitative studies, especially cross-sectional surveys, are more appropriate for population-based surveys while qualitative methods are useful for in-depth explanation in small-scale research-based assessments.

Although various methods can be used for sampling and recruitment, the key strengths of household-based cross-sectional surveys is the representativeness of the population. Although the sizes of samples are usually limited by the budget available for very large surveys, a representative sampling frame is essential for generalization of the survey findings to the population [32]. Inappropriate sampling frames were seen in the studies conducted in Lebanon and Syria [20,26]). In the Lebanon study, which aimed to assess knowledge, attitudes and practice of antibiotic use in the Lebanese population, the sampling frame was the population in the capital city which did not therefore represent the whole population. In the Syrian study, which aimed to provide an insight of the current knowledge and practices regarding antibiotic use among individuals living in the Kalamoon, Syrian Arab Republic, the sampling frame was of households in main streets of two cities which therefore missed some samples for representing the whole population.

We acknowledge that while random sampling is ideal as it properly represents the population, it is time- and resource-consuming. Stratified random sampling and cluster random sampling can be applied to household-surveys as these methods can also achieve representativeness and reduce selection bias. Cluster random sampling is also less costly and feasible; it is a common method used by many studies [33].

Recruiting samples such as adult members or those who have clear understanding of the language used in surveys is critical for ensuring high-quality responses in many surveys. However, specific sampling methods may introduce selection biases, which should be considered before setting inclusion and exclusion criteria.

The high level of non-response rate such as refusals, unreachable households or incomplete data compromises the validity of survey results and conclusions [10].

Although two studies had low response rates, only Vallin M. et al (2016) mentioned this consideration in the discussion section. Various measures can be applied to minimize non-response errors such as making appointments for follow-up interviews for those who were absent on the interview days or using combined user-friendly survey instruments such as face-to-face interviews, telephone, mail or online self-administered surveys [32]. Even where there is high response rate, the non-responders profiles such as those are very high or very lower users of antibiotics; this non-respondent bias can affect the validity of findings about the prevalence of antibiotic use in the population. Almost all studies did not address and describe the profiles of non-responders in their studies which therefore affected the credibility of results. Parameters about non-respondents should be recorded during the field survey and analyzed to verify if the non-responders are similar or dis-similar to the responders.

Two broad methods of questionnaire administration are identified in this review: a) interview survey, either face-to-face or the use of telephone by trained interviewers; and b) self-administration either through postal or internet methods. Using multiple survey methods, when no single method is adequate to address research objective, can minimize the low response rate, prevent coverage, measurement and non-response errors [32]. Each method may have its advantages and disadvantages. For example, self-administered surveys present challenges of interpreting questions as it is “one-way communication” which can introduce measurement error. Face-to-face interviews can prevent measurement bias.

Many studies addressed limitations about coverage errors and measurement errors.

In term of coverage errors, Andre´ M. et al (2010) addressed the fact that 6% of the Swedish population aged 16–75 years did not have a fixed telephone line in 2006. Parimi N. et al (2002) also mentioned that 10% of the households in Trinidad and Tobago did not have telephone service and that 15% of the Telecommunication Services customers have unlisted telephone numbers. However, the limitations of questionnaire administration depend on the context specific to each country.

In relation to measurement errors, Parimi N. et al (2002), Eng JV. et al. (2003) and Barah F. and Goncalves V. (2010) raised concerns about the level of understanding as regard to the questionnaire such as the term ‘antibiotics’ or explanation about illness and treatments. Therefore, some studies reduced these errors by setting criteria to recruit respondents who understood the term ‘antibiotics’ or to those who had used it before. Nevertheless, selection bias should be taken into consideration when studies select based on these specific groups. Interestingly, findings from Widayati A. et al (2012) showed that the characteristics data from groups of responders who were and were not familiar with antibiotics, were not significantly different. It means using this inclusion criterion was useful for ensuring the validity of the questionnaire.

Tailor-made design in line with country contexts is important. For example, in countries with a high prevalence of “polypharmacy” which means using multiple drugs to treat a single ailment or condition at the same time, a careful design is needed to ensure correct interpretation of respondents’ understanding and their ability to distinguish antibiotics from vitamins and analgesic they use. Antibiotics are one of the most common items in polypharmacy, which can cause serious adverse drug events or drug interactions [34]. Additionally, to assess the effectiveness of antibiotic awareness campaigns, the surveys should align with the campaign’s contents as seen in these two studies: Mazińska B. et al (2017) and Haenssgen JM. et al (2018).

With regard to the validity and reliability of measurement, half of these 22 studies did not report testing validity and reliability before finalizing the questionnaire. Although some questionnaires were developed by other studies, the validity and reliability test are still essential because of the difference in population, health systems, culture and terminology for which adaptation to local contexts would be required.

Key findings from these studies showed the differences in prevalence regarding antibiotic use, levels of knowledge of antibiotics and awareness of AMR, frequency and sources of receiving information about antibiotic and its use and AMR. However, there is no study expounding on the outcomes of surveys, including further implications such as impacts on AMR trends. All studies tried to identify the gaps in low levels of knowledge and awareness in terms of contents and characteristics of population in these groups. Furthermore, almost all studies assessed the association between demographics, for example, sex, age, education levels, wealth status with levels of knowledge and awareness, practices, regarding to antibiotic use and AMR except Ahmad H. et al (2013). According to findings, education levels were proven as consensual factors associated with knowledge and awareness while other factors differed depended on each study. Some studies found the significant association between key variables such as exposure to information or campaigns with levels of knowledge and awareness [21], and level of knowledge with level of awareness [16,22,27]. Nevertheless, the relation between levels of knowledge and awareness and antibiotic use remained unclear [11,13] and there were no studies linking the findings with AMR trends. In discussion section, all studies recommended the enhancing of knowledge and awareness from key findings. Most common sources of information are from health workers so they should be key actors in promoting appropriate antibiotic use [14].

Aligned with global action plan on AMR, surveillance on levels of knowledge and awareness can contribute to the design of interventions which can change the population’s behavior on antibiotic use which could potentially lead to a reduction in AMR [35]. Nonetheless, the majority of the reviewed studies encountered limitations in demonstrating an association between knowledge/awareness/practices and the emergence of AMR in the community. Only two studies described the association between knowledge and attitudes, and practices of antibiotic use—showing the association between these factors [11,13]. If a novel antibiotic survey is to be implemented in order to reduce inappropriate antibiotic use, standardized questions on knowledge, awareness and practices on antibiotic use should be focused.

To change pattern of inappropriate uses in the population, it is necessary to have strategies or policies developed based on survey evidences. Experts and academics in the field should discuss and reach consensus on the required questions in the AMR survey module. The critical point is the linkage between levels of knowledge and awareness to behaviors which are influenced by various factors such as access to healthcare or social and cultural aspects [36]. The surveys can be a surrogate measure used to probe into possible causes of the problem. Survey evidence can be used for public advocacy. However, among these studies, only five studies linked their surveys with communication campaigns and evaluate policies [4,6,13,17,28].

The strength of this study is the focus on evaluation of questionnaire tools in household-based cross-sectional surveys. The systematic review contributes to new knowledge about the monitoring of knowledge and awareness of antibiotic use and AMR in two key areas. Firstly, it provides quality assessment of these cross-sectional surveys, which is important for tool development and data collection. We find that AXIS is a useful tool which provides qualitative assessment for the review of survey methodologies [9,10]. Secondly, the previous reviews focused on results of studies; this study fills review gaps by looking at the main contents of the questions that were asked by these 22 survey instruments and associated factors related to knowledge, awareness and practice of antibiotic use and AMR.

However, there remain some limitations. For instance, firstly, despite the authors widened the search strategy as large as possible; it is very likely that some studies had been left out, particularly the gray literature in the archives of domestic universities or research institutes. Secondly, this review was unable to capture the linkage between AMR tools and the actual knowledge and behavior of antibiotic use in the wider population. This issue cannot be addressed by the review; primary data collection through either quantitative survey or qualitative interview is needed. In addition, a more complex review design (for example, realist review) [37] are likely to be beneficial to answering this question. Future systematic reviews that explore the tools in specific subpopulations, such as health professionals, patients, and general populations, are of huge value in the AMR field.

## Conclusion

In response to AMR threats, countries need to assess their population’s knowledge and awareness of antibiotic use and AMR. Valid household-based assessments require clear survey objectives, valid and reliable tools for measurement, representativeness for generalizing the survey findings to the population and minimize sampling and non-sampling biases.

The survey design needs to take into account local contexts and terminologies related to medicines, antibiotics and disease conditions used by the communities, and recruit qualified respondents who can provide accurate responses representing the population. Common questions in existing household-based surveys cover four thematic areas: behavior related to antibiotic use, knowledge and awareness of antibiotic use, knowledge and awareness of AMR and others such as receiving information about antibiotic use and AMR or cross-cutting issues like self-medication.

Countries can learn from previous survey instruments applied by other and avoid mistakes. Accurate survey tools contribute to valid evidence which can be used to inform policies for specific interventions to improve population knowledge and awareness on antibiotics and AMR. The country-specific health system context of access to health services and antibiotics should be taken into account in the design of the survey questionnaire. Identifying levels of knowledge and awareness of antibiotic use and AMR is crucial. Eventually the utmost goals of such surveys would be to enhance the application of this knowledge to target specific target groups as well as to generate public health interventions related to antibiotic use and mitigating AMR.
